# Comparison of Pluripotency, Differentiation, and Mitochondrial Metabolism Capacity in Three-Dimensional Spheroid Formation of Dental Pulp-Derived Mesenchymal Stem Cells

**DOI:** 10.1155/2021/5540877

**Published:** 2021-07-13

**Authors:** Young-Bum Son, Dinesh Bharti, Saet-Byul Kim, Chan-Hee Jo, Eun-Yeong Bok, Sung-Lim Lee, Young-Hoon Kang, Gyu-Jin Rho

**Affiliations:** ^1^Department of Theriogenology and Biotechnology, College of Veterinary Medicine and Research Institute of Life Science, Gyeongsang National University, Jinju, Republic of Korea; ^2^Department of Oral and Maxillofacial Surgery, Changwon Gyeongsang National University Hospital, Gyeongsang National University School of Medicine, Jinju, Republic of Korea

## Abstract

Mesenchymal stem cells (MSCs) are valuable candidates in tissue engineering and stem cell-based therapy. Traditionally, MSCs derived from various tissues have been successfully expanded in vitro using adherent culture plates commonly called as monolayer two-dimensional (2D) cultures. Recently, many studies demonstrated that stemness and multilineage differentiation potential could be enhanced to greater extent when MSCs are cultured as suspended aggregates by means of three-dimensional (3D) culturing techniques. However, there are limited reports on changed mitochondrial metabolism on 3D spheroid formation of MSCs. Therefore, the present study was aimed at investigating the stemness, differentiation potential, and mitochondrial metabolism capacity of 3D dental pulp-derived MSC (DPSC) spheroids in comparison to monolayer cultured DPSCs. We isolated dental pulp-derived MSCs (DPSCs) and successfully developed a 3D culture system which facilitated the formation of MSC spheroids. The cell aggregation was observed after 2 hours, and spheroids were formed after 24 hours and remained in shape for 72 hours. After spheroid formation, the levels of pluripotent markers increased along with enhancement in adipogenic and osteogenic potential compared to 2D cultured control cells. However, decreased proliferative capacity, cell cycle arrest, and elevated apoptosis rate were observed with the time course of the 3D culture except for the initial 24-hour aggregation. Furthermore, oxygen consumption rates of living cells decreased with the time course of the aggregation except for the initial 24 hours. Overall, our study indicated that the short-term 3D culture of MSCs could be a suitable alternative to culture the cells.

## 1. Introduction

Stem cells are valuable cell sources due to their peculiar properties including self-renewability and multilineage differentiation potential [1–5]. Among different types of stem cells, MSCs hold valuable position due to their multilineage differentiation potential and are considered safe because of their nontumorigenic characteristics [1–3]. MSCs are present as heterogonous and rare populations in various tissues such as bone marrow, fat, umbilical cord blood, muscle, Wharton's jelly, and dental tissue [1–3, 6–8]. Therefore, to be used in stem cell-based therapy, isolation and culturing of their homogenous population are highly important. Conventionally, MSCs have been cultured as plastic adherent cells [6, 7]. However, the majority of the studies dealing with two-dimensional (2D) monolayer culturing of the cells have suggested that conventional 2D culturing (which lacks a true in vivo cellular niche) might be unsuitable and results in gradual loss of stemness and differentiation capacity in a time-dependent manner [9–12]. Due to these adversities, concerns about efficacy and safety of MSCs in relation to stem cell therapy have been increased [9–12]. Therefore, it is highly important to pay more attention towards new culturing conditions with special focus on improving the therapeutic effect of MSCs.

Several studies have demonstrated the improved stemness and differentiation potential of MSCs under 3D culture conditions [13, 14]. Under 3D culturing, aggregated MSCs displayed higher expression of pluripotency markers Oct4, Sox2, and Nanog and thereby exhibited higher self-renewability [13, 14]. Further, osteogenic, adipogenic, and neurogenic ability was enhanced in aggregated MSCs in comparison to monolayer adherent cultured cells [15–17]. Moreover, formation of MSC spheroids also resulted in improved functional properties thereby enhancing their therapeutic efficiency by increased secretion of anti-inflammatory cytokines and chemokines [18–20]. Additionally, the genes associated with the cytokine activity, regulation of cell adhesion, receptor binding, growth factor activity, cell communication, response to wounding, extracellular matrix, cell-to-cell signaling, and inflammatory response were upregulated [14].

Despite having enhanced potential in MSC spheroids, several limitations including decreased cell proliferation along with increased apoptotic rate were also reported in the MSC 3D culture system [21–24]. Due to the specific morphology of the spheroid, the cells located in the inner part of the spheroid are deficient in nutrients and oxygen supply and these factors interfere with cell viability and other important parameters [22–24]. Interestingly, in the process of the spheroid culture of MSCs, the mitochondrial membrane potential and ATP production are also decreased which shows effect on the proliferation and apoptosis process [23, 25–27].

Glucose metabolism is a stapling mechanism for the maintenance of tissues. In a condition under sufficient supply of oxygen and nutrients, cells use a large amount of glucose through OXPHOS (oxidative phosphorylation) and pyruvate metabolism through the TCA (tricarboxylic acid) cycle, which occur in mitochondria and finally produce ATPs [28–30]. These processes have a positive effect on cell proliferation and cellular metabolism [28–30]. However, when oxygen and nutrient supply is insufficient, anaerobic glycolysis takes place and ATP production is reduced [30–32]. In connection with these basic mechanisms, several studies reported that cellular apoptosis is greatly influenced by glucose metabolism [30–32]. Therefore, there is an immense need to focus on problems arising due to MSC spheroid formation.

The present study was aimed at evaluating the effect of spheroid formation on the CD marker, proliferation, apoptosis, and osteogenic and adipogenic differentiation capacity of the cultured MSCs. Additionally, changes in the oxygen consumption rate of mitochondria in aggregated cells as compared to monolayer cultured cells were also analyzed.

## 2. Materials and Methods

All chemicals were purchased from Sigma (St. Louis, MO, USA) and media from Gibco (Invitrogen, Burlington, ON, Canada) unless otherwise specified.

### 2.1. Isolation and Culture of Human Dental Pulp-Derived MSCs

All patients were provided with informed consent for the collection of dental pulp tissues, in accordance with the approved medical guidelines of the Ethics Committee of Gyeongsang National University Hospital (GNUH-IRB-2018-11-002-001). The dental pulp tissues were acquired from the extracted immature wisdom teeth of six patients (three men and three women) with an average age of 18.5 ± 2.3 yrs. The isolation of MSCs from dental pulp tissues was conducted following previous reports [2, 33]. Briefly, after being washed with Dulbecco's phosphate-buffered saline (DPBS) containing 1% penicillin/streptomycin (10,000 IU and 10,000 *μ*g/ml, respectively), tissues were minced into 1 to 3 mm^2^ explants and digested in DPBS containing collagenase type I (1 mg/ml) at 37°C. Furthermore, Dulbecco's modified Eagle's medium (DMEM) containing 10% fetal bovine serum (FBS) was added in the digested tissue samples, and samples were filtered through 40 *μ*m nylon cell strainers (Falcon®, Franklin, NJ, USA). The filtered cell pellets were centrifuged at 300 × *g* for 5 minutes and cultured in DMEM supplemented with 10% FBS, 1% (*v*/*v*) nonessential amino acids (NEAA; Invitrogen, Carlsbad, California, USA), and 1% penicillin/streptomycin in a humidified incubator at 5% CO_2_. At 80 to 90% confluence, the cells were detached with 0.25% trypsin EDTA solution and further subcultured. The media were changed every 48 to 72 hours during the culture. DPSCs at passage 3 were used for the whole experimentation in the following way. DPSCs were cultured at 80% confluence of passage 3 for homogenization; some of the cells were used for spheroid culture. Another DPSCs at passage 3 were resuspended further for the 2D control group, which was reharvested for additional 72 hours at 80% confluence to adjust the timeline to the 3D culture groups.

### 2.2. Spheroid Culture of Dental Pulp Stem Cells (DPSCs)

To make DPSC spheroids, 3D culture was performed using a StemFIT 3D dish (microFIT, Seongnam, Korea) following the manufacturer's protocol. Briefly, spheroid-forming dishes were placed on the culture plate followed by gently washing with 70% ethanol for few times to remove the air bubbles in the wells. A total of 1 × 10^6^ DPSCs mixed with 1 ml culture media were carefully added into the spheroid-forming 3D wells without forming bubbles, and after 10 minutes, floating cells were removed. The cells were cultured in an incubator for 72 hours and sampled for analysis every 24 hours. The media were changed every 24 hours during the culture.

### 2.3. Cell Proliferation

To evaluate the cell conditions, we performed a population of doubling time (PDT) assay. A total of 2 × 10^3^ 2D and 3D cultured DPSCs were cultured in an incubator. At 24-hour intervals, cells were counted using a hemocytometer for 72 hours. The PDT was calculated with the equation, PDT = log2 × *T*/(logNC–logNI), where *T* is the cell culture time, logNC is the cultured cell number, and logNI is the initial cell number.

### 2.4. Cell Surface Marker Analysis

The expression of MSC-specific positive (CD44, CD73, CD90, and CD105) and negative (CD34 and CD45) markers was evaluated using the BD FACSVerse™ instrument (BD Biosciences, Franklin Lakes, NJ, USA) (Table 1). We obtained single-cell suspensions from the monolayer (2D) and spheroid cultured (3D) DPSCs by trypsinization. After that, the cells were stained with fluorescein isothiocyanate- (FITC-) conjugated anti-CD34 (mouse monoclonal, BD Biosciences, Franklin Lakes, NJ, USA), CD45 (mouse monoclonal, BD Biosciences, Franklin Lakes, NJ, USA), CD44 (mouse monoclonal, BD Biosciences, Franklin Lakes, NJ, USA), CD73 (mouse monoclonal, BD Biosciences, Franklin Lakes, NJ, USA), and CD90 (mouse monoclonal, BD Biosciences, Franklin Lakes, NJ, USA) for 1 hour. Analysis of CD105 expression was performed by treating the cells with a primary antibody (mouse monoclonal, Santa Cruz Biotechnology, Dallas, TX, USA) for 1 h followed by staining with an FITC-conjugated secondary antibody (Santa Cruz Biotechnology, Dallas, TX, USA). A total of 1 × 10^4^ cells were measured by flow cytometry. All antibodies were diluted to 1 : 100 with 1% bovine serum albumin (BSA). For isotype control, FITC mouse IgG (BD Biosciences, Franklin Lakes, NJ, USA) was used.

### 2.5. Cell Cycle Analysis and Apoptosis Analysis

For confirming the cell conditions, cell cycle analysis was carried out following a previous report [6]. DPSCs cultured in 2D and 3D were fixed with 70% ethanol at 4°C overnight. After that, cells were washed with DPBS and stained with propidium iodide solution (10 *μ*g/ml, PI) for 15 minutes. Furthermore, cell cycle events, i.e., G0/G1, S, and G2/M, were measured by flow cytometry.

To detect the rate of apoptosis, the FITC-Annexin V Apoptosis Detection Kit (Invitrogen, USA) was used following the manufacturer's instructions. In brief, 2D and 3D cultured DPSCs were washed with DPBS, and 1x annexin-binding buffer (100 *μ*l) was treated by staining with Alexa Fluor annexin V and PI for 15 minutes in an incubator. The stained cells were resuspended with annexin-binding buffer and analyzed by flow cytometry.

### 2.6. In Vitro Osteoblast and Adipocyte Differentiation

To investigate the in vitro differentiation into mesenchymal lineages, 2D and 3D cultured DPSCs were induced into osteocytes and adipocytes using suitable culture conditions as previously reported [6, 7]. Briefly, cells were seeded into 6-well culture plates (Thermo, Suzhou, Jiangsu, China). For osteogenic differentiation, cells were cultured in DMEM containing 10% FBS, 10 nM dexamethasone, 50 *μ*g/ml ascorbic acid, and 10 mM sodium *β*-glycerophosphate for 21 days. Furthermore, mineralization and calcium deposition were detected with the help of von Kossa and Alizarin red staining followed by evaluation of osteocyte-specific gene expression.

For adipogenic differentiation, cells were cultured in DMEM containing 10% FBS, 100 *μ*M indomethacin, 10 *μ*M insulin, and 1 *μ*M dexamethasone for 21 days, and successful differentiation was evaluated by the accumulation of lipid droplets by using Oil red O staining. The extent of differentiation was further confirmed by the expression of adipocyte-specific genes.

### 2.7. Real-Time Quantitative Polymerase Chain Reaction (RT-qPCR) Analysis

The expression of pluripotency and osteoblast- and adipocyte-specific genes was analyzed using real-time quantitative polymerase chain reaction analysis (RT-qPCR) as previously described in the protocol with minor modifications [34]. Total RNA was extracted from the 2D and 3D cultured MSCs and induced osteoblast and adipocyte groups using the easy-spin total RNA Extraction Kit (iNtRON, Seongnam, Korea) and quantified using a Nanodrop 1000 spectrophotometer (Thermo Fisher Scientific, Waltham, MA, USA). After that, complementary DNA (cDNA) was synthesized from purified RNA (2 *μ*g) by using the HisenScript RT PreMix kit (iNtRON, Seongnam, Korea) with 10 *μ*M OligodT primer at 42°C for 50 minutes. The RT-qPCR was carried out using a Rotor-Gene Q cycler (Qiagen, Hilden, Germany) and RealMOD™ Green AP 5x qPCR mix (iNtRON, Seongnam, Korea) containing 200 nM of forward and reverse primers with 50 ng/*μ*l cDNA samples. The RT-qPCR setting included denaturation at 95°C for 60 s followed by 40 cycles of 95°C for 10 min, 60°C for 6 s, and 72°C for 4 s. Gene expression was analyzed for the mRNA levels of an internal control gene, tyrosine 3-monooxygenase/tryptophan 5-monooxygenase activation protein, zeta polypeptide (YWHAZ). All samples were analyzed in triplicate. The list of primers is mentioned in Table 2.

### 2.8. Oxygen Consumption Rate Analysis

Seahorse XFp Cell Mito Stress Tests (Seahorse, Agilent Technologies, Santa Clara, CA) were performed following the manufacturer's protocol. At 24 hours prior to the assay, 2D and 3D cultured DPSCs were plated and cultured at 50,000 cells/well in a Seahorse XF24 plate (Seahorse; Bioscience, Billerica, MA, USA). After that, XF24 media were supplemented with 2 *μ*M rotenone, 1 *μ*M FCCP, and 1 *μ*g/ml oligomycin. Treatment with the drugs into the medium occurred at the time points specified. The oxygen consumption rate (OCR) was analyzed using a Seahorse Bioscience XF24 Extracellular Flux Analyzer. The basal respiration rate was calculated before oligomycin treatment, and proton leak was measured after oligomycin treatment. The values of ATP production were measured through the difference between basal respiration and proton leak, and after FCCP treatment, spare respiratory capacity was confirmed through the difference between maximal respiration and ATP production.

### 2.9. Statistical Analysis

Data analysis was performed by one-way analysis of variance (ANOVA) using SPSS (version 15, SPSS Inc., Chicago, Illinois), and the graph was prepared with GraphPad Prism (version 4.0) software. Tukey's test was conducted for between-group comparisons. Data were represented as mean ± standard deviation (SD), and *p* < 0.05 was considered statistically significant.

## 3. Results

### 3.1. Formation of DPSC Spheroids in 3D Culture

**Figure 1:**
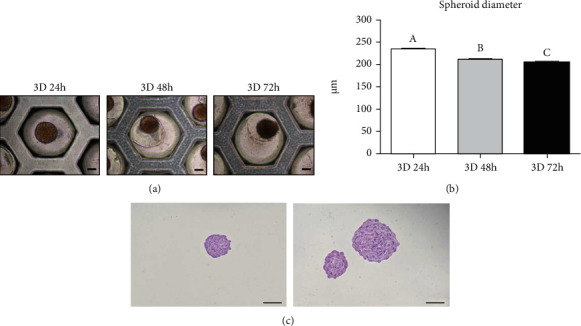
Formation of spheroids using DPSCs. (a) The aggregation of DPSCs into spheroids in 3D culture conditions (scale bar = 100 *μ*m). (b) The DPSC spheroids became compact, and the diameter decreased with the time course of the 3D culture. Different superscripts (a to c) represent a significant (*p* < 0.05) difference. (c) H&E staining of the spheroid section from the 24-hour cultured group (scale bar = 200 *μ*m).

For the generation of spheroids, DPSCs were cultured in StemFIT culture dishes. After 24 hours, many small aggregated cells were confirmed and one large spheroid was constructed in each well (Figure 1(a)). Once spheroids formed, it persisted up to 72 hours. The diameter of the spheroid was decreased with the time course of the 3D culture. The average fold change of the diameter of spheroids under 3D culture conditions for 24, 48, and 72 hours was 235.41 ± 2.93, 212.56 ± 1.82, and 204.95 ± 1.85 *μ*m, respectively (Figure 1(b)). H&E staining of spheroid sections showed that spheroid was compact throughout with small single cells evenly distributed (Figure 1(c)).

### 3.2. Expression of Pluripotent Markers in DPSC Spheroids

**Figure 2:**
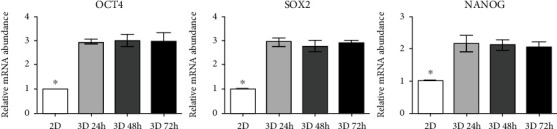
Spheroid DPSCs expressed a high level of pluripotent markers. Monolayer cultured cells (2D) showed significantly decreased pluripotent gene (*OCT4*, *SOX2*, and *NANOG*) expression as compared to 24-hour 3D cultured (3D 24 h), 48-hour 3D cultured (3D 48 h), and 72-hour 3D cultured (3D 72 h) cells. Asterisk (∗) represents a significant (*p* < 0.05) difference.

To confirm the effect of 3D cultivation on the stemness of DPSCs, we analyzed the expression of pluripotency markers in 2D and 3D cultured DPSCs by RT-qPCR (Figure 2). Our results demonstrated that the expression of Oct4, Sox2, and Nanog was significantly (*p* < 0.05) increased in all 3D cultured DPSC groups when compared to 2D cultured DPSCs. However, there was no significant difference in the expression of pluripotency markers depending on the time in 3D cultivation.

### 3.3. Osteogenic and Adipogenic Differentiation Potential of 2D and 3D Cultured DPSCs

**Figure 3:**
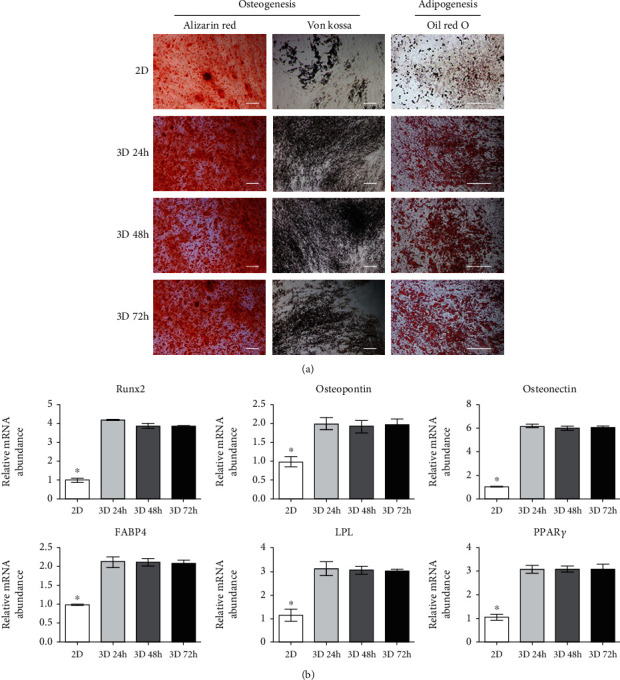
In vitro osteogenic and adipogenic differentiation capacity of DPSCs. (a) The adipocyte differentiation of DPSCs was evaluated by Oil red O staining of lipid droplets. Osteogenesis was evaluated by Alizarin red and von Kossa staining of mineralization and calcium deposition (scale bar = 200 *μ*m). The 2D cultured DPSC group showed to be most weakly stained with Oil red O, Alizarin red, and von Kossa. (b) Expression of osteoblast- (*Runx2*, *osteopontin*, and *osteonectin*) and adipocyte- (*FABP4*, *LPL*, and *PPARγ*) specific genes in induced 2D and 3D cultured DPSC groups. All osteogenesis- and adipogenesis-specific markers were significantly increased in induced 3D cultured DPSC groups as compared to 2D cultured DPSCs. However, no difference was confirmed among the 24-hour 3D cultured (3D 24 h), 48-hour 3D cultured (3D 48 h), and 72-hour 3D cultured (3D 72 h) cells. *YWHAZ* was used as an internal control. Asterisk (∗) represents a significant (*p* < 0.05) difference.

To evaluate the osteoblast and adipocyte differentiation potential of both 2D and 3D cultured DPSCs, cells were induced to osteoblast and adipocyte lineages under lineage-specific culture conditions. Osteoblast-differentiated cells exhibited strong Alizarin red and von Kossa staining from 3D cultured DPSCs in comparison to 2D cultured cells (Figure 3(a)) and confirmed mineralized nodule formation. On the other hand, 3D differentiated adipocytes showed high Oil red O staining when compared to 2D differentiated adipocytes (Figure 3(a)) and hence confirmed accumulation of intracellular lipid droplets. RT-qPCR results followed the same pattern where high gene expression (both osteocyte and adipocyte) was shown by 3D differentiated cells than 2D differentiated cells (Figure 3(b)). The expression of Runt-related transcription factor 2 (*Runx2*), *osteopontin*, and *osteonectin* was significantly increased in the differentiated cells derived from 3D cultured DPSCs as compared to those derived from 2D cultured DPSCs (Figure 3(b)). However, there was no significant difference in the expression of osteoblast-specific genes depending on the time in 3D cultivation. In case of adipocyte differentiation, expression of fatty acid-binding protein 4 (*FABP4*), lipoprotein lipase (*LPL*), and peroxisome proliferator-activated receptor (*PPARγ*) was significantly increased in the differentiated cells derived from 3D cultured DPSCs as compared to those derived from 2D cultured DPSCs (Figure 3(b)). Like osteocyte differentiation, there was no significant difference in the expression of adipocyte-specific genes depending on the time in 3D cultivation.

### 3.4. Cell Proliferation, Cycle, Cellular Apoptosis, and Expression of Cell Surface Marker of 2D and 3D Cultured DPSCs

**Figure 4:**
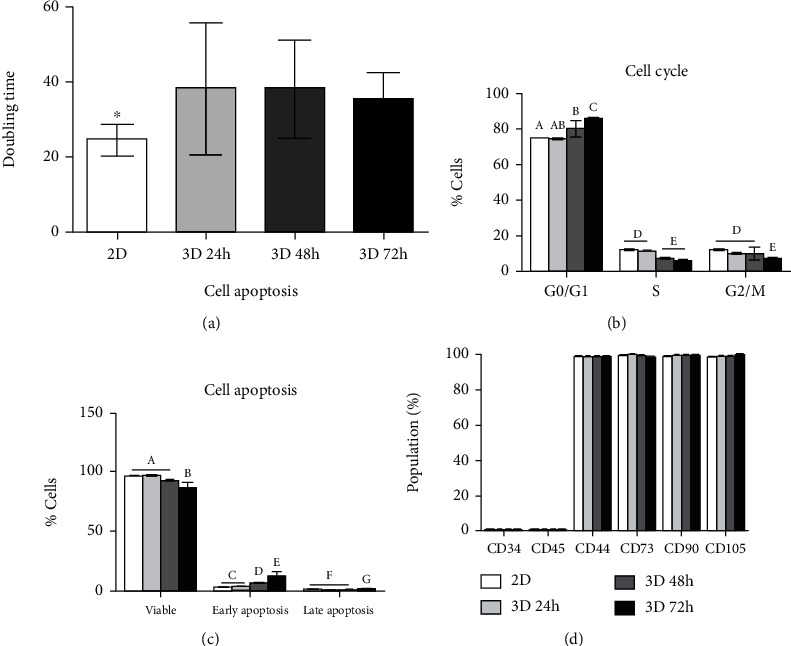
The characteristics of 2D and 3D cultured DPSCs with the time course of the 3D culture. (a) Analysis of cell proliferation by population doubling time (PDT). The 3D cultured DPSCs revealed decreased proliferation capacity as compared to 2D cultured DPSCs. Asterisk (∗) represents a significant (*p* < 0.05) difference. (b) Cell cycle analysis showed that cell arrest was increased and DNA replication was increased in 3D cultured DPSCs (*p* < 0.05). (c) Similarly, cellular apoptosis was also increased, and the portion of the viable cell was decreased in 3D cultured DPSCs with the time course. (d) There was no difference in the CD marker profiling among the 2D and 3D cultured DPSCs. Different superscripts (a to g) represent a significant (*p* < 0.05) difference.

The cell viability and characteristics were analyzed under 3D cultivation. The population doubling time (PDT) was measured every 24 hours in 3D cultured DPSCs (Figure 4(a)). The 3D cultured DPSCs showed significantly increased doubling time as compared to 2D cultured DPSCs. Additionally, cell cycle analysis revealed that the portion of the G0/G1 phase indicating cell arrest was significantly increased and the S phase (which corresponds to DNA replication) was significantly decreased in 3D cultured DPSCs as compared to 2D cultured DPSCs (Figure 4(b)). Cellular apoptosis was evaluated using the annexin V/PI assay (Figure 4(c)). The portion of viable, early, and late apoptotic cells was similar in 2D and 24-hour 3D cultured DPSCs. Flow cytometric analysis showed that 2D and 3D cultured MSCs were positive for the mesenchymal stem cell markers (CD44, CD73, CD90, and CD105) and were negative for the hematopoietic stem cell markers (CD34 and CD45) (Figure 4(d)). There was no significant difference in the CD marker expression in the 2D and 3D cultured DPSCs (Figure 4(d)).

### 3.5. Oxygen Consumption Rate Analysis

**Figure 5:**
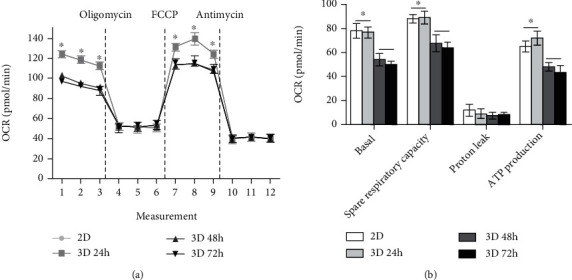
The oxygen consumption rate of 2D cultured and 3D cultured DPSCs. (a) The 48-hour and 72-hour 3D cultured DPSCs (3D 48 h and 3D 72 h, respectively) revealed decreased mitochondrial respiration compared to 2D and 24-hour 3D cultured DPSCs (2D and 3D 24 h, respectively). (b) The quantification of basal respiration, spare respiratory capacity, and ATP production was decreased in 3D 48 h and 3D 72 h as compared with 2D and 3D 24 h. The values of data in OCR represented mean ± SD (*n* = 10 wells in independent experiments). Asterisk (∗) indicates statistical differences between groups (*p* < 0.05).

OCR was used to assess cellular energetics in 3D culture conditions compared to 2D culture conditions. Our results showed that basal OCR, which was an indicator of oxidative phosphorylation (OXPHOS), was significantly decreased in 48-hour and 72-hour 3D cultured DPSCs as compared to 2D cultured and 24-hour 3D cultured DPSCs (Figure 5). After treatment with oligomycin, which inhibited the F0 ATP complex, the OCR was rapidly decreased in all groups (Figure 5(a)). After the addition of FCCP, mitochondrial respiration was uncoupled and OCR was increased in all groups (Figure 5(a)). Our results revealed that 48-hour and 72-hour 3D cultured DPSCs showed significantly lower basal respiration, spare respiratory capacity, and ATP production when compared to 2D and 24-hour 3D cultured DPSCs (Figure 5(b)).

## 4. Discussion

Many studies have reported the self-renewability and multidifferentiation potential of MSCs [1–3]. Conventional MSC culture involved adherent culture conditions; however, it was demonstrated that monolayer culture conditions exhibit a nonphysiological environment and hence lack some of the in vivo characteristics [18, 20, 21]. Following in vitro culture passage, MSCs showed several changes in characteristics, including reduced self-renewal, multilineage differentiation capacity, expression of pluripotency markers, and glucose metabolism [1–3, 25]. The microenvironment of culture conditions also plays an important role in the decision of cell fate [23, 25, 35] as 2D culture conditions did not support a proper microenvironment for MSCs [9–12]. Therefore, many studies focused on maintaining and enhancing stemness along with pluripotent nature of MSCs by using 3D culture systems [13, 14].

In 3D culture conditions, MSCs aggregate with each other to form spheroids and provide cellular niches similar to in vivo conditions [23, 25, 35]. These aggregated MSCs showed enhanced multilineage differentiation potential, homing ability, secretion of growth factors, and paracrine factor production [23, 25, 35]. Furthermore, studies revealed that direct cell-to-cell interaction in the 3D culture conditions could provide regulation of various biological characteristics such as shape and size of cells, oxygen and nutrient supply, and signal transduction [36, 37]. Therefore, 3D culturing was suggested as an efficient alternative method [36]. Despite such observations, long-term culture of MSC spheroids showed agglomeration of cells and resulted in the formation of necrotic centres due to the limited nutrients and oxygen supply into the spheroid centre. They showed ongoing apoptosis and reduced proliferation capacity, along with decreased mitochondrial membrane potential and ATP production [21–25]. Therefore, it was important to investigate the changes, which occur during 3D culture systems. Keeping these necessities in mind, with the help of the microwell chip, homogenous MSC spheroids (with similar size and shape) were formed under 3D culturing conditions.

It is well known that pluripotency markers play an important role in regulating the self-renewability and pluripotent nature of the MSCs [38]. Several studies showed that the expression of these markers increased in MSCs under 3D culture conditions when compared to 2D culture conditions [13, 14]. Consistent with previous reports, our results displayed enhanced Oct4, Sox2, and Nanog expression in 3D cultured DPSCs compared to 2D cultured cells; however, no differences were seen among the time course of 3D culture. These results implicate that MSCs maintain their pluripotent nature irrespective of time course duration under 3D culturing.

As previously mentioned, the spheroid culture of MSCs increases osteogenic and adipogenic differentiation potential [15–17]. Similarly, in this study, cytochemical staining and lineage-specific marker expression revealed improvements in osteocyte and adipocyte differentiation potential from induced DPSCs cultured in 3D conditions in comparison to 2D cultured cells (Figure 3). Interestingly, lineage differentiation capacity (osteocyte and adipocyte) was not different in 3D cultured DPSCs regardless of the 3D culture time. Overall results concluded that the cells cultured for 24 hours under 3D conditions sufficiently improved pluripotency as well as osteogenic and adipogenic differentiation capacity.

The cellular senescence and proliferation capacity constitute other important factors that need evaluation under 3D culture conditions. Previous studies reported that long-term culture of MSCs under 3D culture conditions causes decreased cell proliferation while enhancing the apoptosis rate due to cellular senescence [22–24]. Similar findings observed in the present study, where DPSCs cultured under 3D conditions for more than 24 hours, displayed significant reduction in the cell proliferation rate when compared to 2D cultured DPSCs (Figure 4(a)). Additionally, the G0/G1 phase indicating cell arrest was significantly increased and the S phase was significantly decreased following 3D culture conditions as compared to monolayer cultured DPSCs (Figure 4(b)). These results implicated that the G0/G1 and S phase gradually changed with the progression of time. The portion of viable cells under 3D culture conditions for 24 and 48 hours was not different as compared to 2D culture conditions. Additionally, the ratio of early and late apoptosis in DPSC under 3D culture conditions for 24 hours was not significantly different from those under 2D cultured DPSCs. Therefore, we confirmed that the DPSC spheroid with the time course of the 3D culture revealed reduced cell viability and increased early and late apoptosis.

Cells continuously maintain a balance between proliferation and apoptosis in the body by an important mechanism, i.e., homeostasis, which is known for regulating the cellular metabolism [23, 25]. The growth of cells is mainly regulated by OXPHOS occurring in the mitochondria [29]. In a study by Tsai and colleagues, it was reported that culturing of aggregated MSCs for 7 days resulted in decreased ATP production and reduced mitochondrial membrane potential [23]. They further demonstrated that the changes in cellular metabolism and mitochondrial depolarization were closely related to cellular proliferation and apoptosis [23]. Consistent with these findings, our study displayed lower basal respiration, spare respiration capacity, and reduced ATP production from 48- and 72-hour 3D cultured DPSCs as compared to others. Overall, it is evident that DPSCs cultured under 3D conditions for more than 24 hours (i.e., 48 and 72 hours in our case) result in decreased cellular metabolism in a similar pattern to that of the cell cycle and apoptosis which occurred due to problems associated with oxygen and nutrient supply under continued prolonged MSC 3D culture.

## 5. Conclusions

In conclusion, homogenous DPSC spheroids were successfully constructed with the help of the microwell chip, and analysis was performed until cells migrated from aggregated DPSCs. DPSCs formed spheroids within 24 hours of 3D culture condition, which not only enhanced the expression of pluripotency markers but also improved osteogenic and adipogenic differentiation potential. It is highly important to notice that prolonged 3D culturing (for more than 24 hours) resulted in undesirable outcomes in the form of cell cycle arrest, cellular apoptosis, and reduced ATP production along with decreased OCR. Interestingly, 24-hour 3D cultured DPSCs shared similar characteristics when compared to monolayer culture conditions. Taken together, we demonstrated that efficient DPSC spheroids could be constructed through short-term 3D culture for 24 hours.

## Figures and Tables

**Table 1:** Lists of antibodies and their amount in immunocytochemistry analysis.

Antibody	Company	Amount
FITC mouse IgG, isotype control	BD Pharmingen™	0.5 mg/ml
FITC mouse anti-human CD34	BD Pharmingen™	0.5 mg/ml
FITC mouse anti-human CD45	BD Pharmingen™	0.5 mg/ml
FITC rat anti-human CD44	BD Pharmingen™	0.5 mg/ml
Mouse anti-human CD73	BD Pharmingen™	0.5 mg/ml
FITC mouse anti-human CD90	BD Pharmingen™	0.5 mg/ml
Mouse monoclonal CD105	Santa Cruz Biotechnology	200 *μ*g/ml
FITC goat anti-mouse IgG	Santa Cruz Biotechnology	0.5 mg/ml

**Table 2:** Lists of primers used in RT-qPCR.

Gene name	Primer sequence	Product size (bp)	Anneal. temp (°C)	Reference
OCT4	F: ACTATCATTGATGCCCCAGGAC R: ACACCAGCAGCAACAATCAG	128	60	NM_021130.3
SOX2	F: CACCCACAGCAAATGACAGC R: GTCCCCCAAAAAGAAGTCCAG	120	60	NM_021130.3
NANOG	F: TGCAACCTGAAGACGTGTG R: TGCAACCTGAAGACGTGTG	153	60	NM_024865.2
FABP4	F: TGAGATTTCCTTCATACTGG R: TGGTTGATTTTCCATCCCAT	128	60	NM_001442.2
LPL	F: AGACACAGCTGAGGACACTT R: GCACCCAACTCTCATACATT	137	60	NM_001442.2
PPAR*γ*	F: TTGCTGTCATTATTCTCAGT R: GAGGACTCAGGGTGGTTCAG	124	60	AB565476.1
Runx2	F: CCTTGGGAAAAATTCAAGCA R: AACACATGACCCAGTGCAAA	181	60	NM_001015051
OPN (osteopontin)	F: CATCACCTCACACATGGAAAGC R: CTGACTCGTTTCATAACTGTCC	115	60	NM_001251830.1
ON (osteonectin)	F: GTGCAGAGGAAACCGAAGAG R: AAGTGGCAGGAAGAGTCGAA	170	60	J03040.1
YWHAZ	F: ACGAAGCTGAAGCAGGAGAAG R: TTTGTGGGACAGCATGGATG	111	60	BC108281.1

## Data Availability

Data access can be requested on demand with the corresponding author.
